# Impact of the peripheral blood inflammatory indices and modified nomogram-revised risk index on survival of Extranodal Nasal-Type Natural Killer/T-Cell lymphoma

**DOI:** 10.3233/CBM-230067

**Published:** 2024-01-05

**Authors:** Qing Hou, He Li, Yu Liang, Ningning Yao, Xin Cao, Jianting Liu, Bochen Sun, Peixin Feng, Wenjuan Zhang, Jianzhong Cao

**Affiliations:** a Department of Radiotherapy, Shanxi Province Cancer Hospital/Shanxi Hospital Affiliated to Cancer Hospital, Chinese Academy of Medical Sciences/Cancer Hospital Affiliated to Shanxi Medical University, Taiyuan, Shanxi, China; b Department of Pathology, Shanxi Province Cancer Hospital/Shanxi Hospital Affiliated to Cancer Hospital, Chinese Academy of Medical Sciences/Cancer Hospital Affiliated to Shanxi Medical University, Taiyuan, Shanxi, China

**Keywords:** Extranodal NK-T-Cell lymphoma, nasal type, biomarker, inflammations, survival

## Abstract

**BACKGROUND:**

At present, peripheral blood markers are easily accessible information and clinically valuable prognostic indicators in extranodal nasal-type natural killer/T-cell lymphoma (ENKTCL). Nevertheless, the role of its comprehensive score in ENKTCL remains to be determined.

**OBJECTIVE:**

Therefore, this study aimed to investigate the prognostic effect of the peripheral inflammation score on ENKTCL.

**METHODS:**

The retrospective study included 183 patients with ENKTCL. Univariate Cox regression analyses and least absolute shrinkage and selection operator (LASSO) Cox regression were used to construct the inflammation-related prognostic index named Risk. Univariate and multivariate Cox regression analyses and regression adjustment with propensity score matching (PSM) were used to evaluate the prognostic ability of risk. The performance of the modified nomogram-revised risk index (NRI) by integrating risk was evaluated with the area under the time-dependent receiver operating characteristic (ROC) curve (AUC), decision curve analysis (DCA), and integrated Brier score (IBS).

**RESULTS:**

The risk cut-off value, constructed by the lymphocyte count, platelet count, albumin level, LMR, and PNI, was -1.3486. Before PSM, multivariate analysis showed that risk was significantly associated with OS (HR = 2.577, 95% CI = 1.614–4.114, P< 0.001) and PFS (HR = 2.679, 95% CI = 1.744–4.114, P< 0.001). After PSM adjustment, risk was still an independent factor for OS (HR = 2.829, 95% CI = 1.601–5.001, P< 0.001) and PFS (HR = 2.877, 95% CI = 1.735–4.770, P< 0.001). With the NRI, the modified NRI by integrating risk increased the AUC and clinical net benefit and decreased the IBS.

**CONCLUSIONS:**

Risk is an easily accessible and inexpensive indicator that may be used as a prognostic marker and could improve NRI predictive power in patients with ENKTCL.

## 1. Introduction

Extranodal Nasal-Type Natural Killer/T-Cell Lymphoma (ENKTCL) is an aggressive extranodal lymphoma of natural killer (NK)-cell or T-cell lineage lymphoma characterized by vascular damage and destruction, prominent necrosis, a cytotoxic phenotype, and is associated with Epstein-Barr virus [1]. ENKTCL is more prevalent among Asian, Mexican, Central American, and South American indigenous populations. ENKTCL is more common in males than females, with a median age of 44–54 years at diagnosis [2].

The most common prognostic scores of ENKTCL include the Korean Prognostic Index (KPI), the International Prognostic Index (IPI), and the prognostic index of natural killer lymphoma (PINK) [3, 4, 5, 6]. However, these prognostic scores still have some limitations in clinical application. They cannot always accurately predict the prognosis, cannot help doctors customize the initial therapy, and cannot identify risk groups that are beneficial to prognosis in early patients [7]. Yang et al. [8] developed and validated an easily used nomogram-revised risk index (NRI) that stratified patients based on age, Ann Arbor stage, Eastern Cooperative Oncology Group (ECOG) scores, lactate dehydrogenase (LDH) levels, and primary tumour invasion (PTI). The NRI can evaluate the outcome more accurately than the KPI, IPI, and PINK. The risk-adaptive therapy proposed based on its conclusions can effectively identify patients who will benefit from radiotherapy followed by chemotherapy [7, 8, 9, 10].

However, the risk factors for NRI did not involve inflammatory biomarkers. Previous studies have shown that inflammation is closely related to the development and prognosis of malignant tumours and has been well validated in central nervous system lymphoma, small cell lung carcinoma, pancreatic cancer, and others [9, 10, 11]. Albumin, monocytes, lymphocytes, platelets, neutrophil-to-lymphocyte ratio (NLR), derived neutrophil-to-lymphocyte ratio (dNLR), platelet-to-lymphocyte ratio (PLR), lymphocyte-to-monocyte ratio (LMR), and prognostic nutritional index (PNI) have been reported to be associated with the prognosis of ENKTCL [12, 13, 14, 15]. Although these inflammatory biomarkers are essential, they provide limited predictive performance when evaluated separately.

This study aimed to evaluate the association between inflammatory biomarkers and ENKTCL patient prognosis and construct a peripheral blood inflammatory index named Risk. Furthermore, we assessed an additional improvement of combined Risk to NRI prediction performance.

## 2. Patients and methods

### 2.1 Patients

A total of 183 patients with a pathological diagnosis of ENKTCL admitted to Shanxi Provincial Cancer Hospital from January 2002 to December 2018 were retrospectively reviewed. The inclusion criteria were the same as those described in our previous study [11]. This study was approved by the Ethics Committee of Shanxi Province Cancer Hospital, Shanxi Hospital Affiliated to Cancer Hospital, Chinese Academy of Medical Sciences, and Cancer Hospital Affiliated to Shanxi Medical University. The Review Committee exempted informed consent requirements.

### 2.2 Laboratory measurement

The laboratory indicators and their derived indicators included albumin, monocyte, lymphocyte, platelet, NLR, dNLR, PLR, LMR, and PNI within one week before treatment. The compound prognostic scores were calculated by Eq. (2.2).

𝑁𝐿𝑅=𝑛𝑒𝑢𝑡𝑟𝑜𝑝ℎ𝑖𝑙/𝑙𝑦𝑚𝑝ℎ𝑜𝑐𝑦𝑡𝑒

𝑑𝑁𝐿𝑅=𝑛𝑒𝑢𝑡𝑟𝑜𝑝ℎ𝑖𝑙/(𝑙𝑒𝑢𝑘𝑜𝑐𝑦𝑡𝑒-𝑛𝑒𝑢𝑡𝑟𝑜𝑝ℎ𝑖𝑙)

(1) 𝑃𝐿𝑅=𝑝𝑙𝑎𝑡𝑒𝑙𝑒𝑡/𝑙𝑦𝑚𝑝ℎ𝑜𝑐𝑦𝑡𝑒

𝐿𝑀𝑅=𝑙𝑦𝑚𝑝ℎ𝑜𝑐𝑦𝑡𝑒/𝑚𝑜𝑛𝑜𝑐𝑦𝑡𝑒

𝑃𝑁𝐼=𝑎𝑙𝑏𝑢𝑚𝑖𝑛+5∗𝑙𝑦𝑚𝑜𝑝ℎ𝑜𝑐𝑦𝑡𝑒

### 2.3 Statistics

Overall survival (OS) was defined as the time from the date of diagnosis to the date of any cause of death or final follow-up. Progression-free survival (PFS) was measured from the date of diagnosis to the date of first recurrence, progression, death, or last follow-up. Maximally selected rank statistics determine the optimal cut-off value of continuous variables. Variables with a P value < 0.05 in univariate Cox regression analysis were included in the least absolute shrinkage and selection operator (LASSO) Cox regression analysis to construct Risk [16]. Chi-square or Fisher’s exact test was used for categorical variables. Propensity score matching (PSM) was performed to adjust the differences between the low- and high-risk groups. Univariate and AIC-based multivariate Cox regression analyses were used to evaluate the prognostic ability of Risk before and after PSM. Kaplan-Meier and log-rank tests were used to plot survival curves and detect the survival differences in stratified risk before and after PSM. The area under the time-dependent receiver operating characteristic (ROC) curve (AUC), decision curve analysis (DCA), and integrated Brier score (IBS) were used to evaluate the performance of the modified NRI, which combined Risk. The overall reclassification improvement was evaluated by the integrated discrimination improvement (IDI), continuous-net reclassification improvement (NRI), and median improvement in risk score. 

## 3. Results

### 3.1 Patient characteristics

The clinical characteristics of all patients are summarized in Table 1. The median age of the patients was 46 years (range 9–81 years). The number of patients receiving radiotherapy and L-asparaginase (L-ASP) was 61 and 83, respectively. The median OS was 137 months, and 86 patients (47.0%) died during the follow-up period. The 3-year and 5-year OS rates were 56.7% and 51.7%, respectively. The 3-year and 5-year PFS were 50.6% and 43.3%, respectively.

**Table 1:** Distribution of clinical characteristics of patients with ENKTCL

Characteristics	Number of patients (%)
Age	
⩽ 60	147 (80.33%)
> 60	36 (19.67%)
Gender	
Male	144 (78.69%)
Female	39 (21.31%)
ECOG	
0–1	147 (80.33%)
⩾ 2	36 (19.67%)
Ann arbor stage	
I–II	141 (77.05%)
III–IV	42 (22.95%)
B symptoms	
Yes	66 (36.07%)
No	117 (63.93%)
No. of extranodal sites	
< 2	158 (86.34%)
⩾ 2	25 (13.66%)
LN	
Yes	66 (36.07%)
No	117 (63.93%)
Primary site	
UADT	169 (92.35%)
Non-UADT	14 (7.65%)
Treatment	
RT alone	14 (7.65%)
CT alone	61 (33.33%)
CRT	108 (59.02%)
CT regimen	
L-Asp-based	83 (45.36%)
Gemcitabine	28 (15.30%)
CHOP or CHOP-like	39 (21.31%)
Others	19 (10.38%)

*UADT*, upper aerodigestive tract NK/T-cell lymphoma; *LN*, Regional lymph node involvement; *RT*, radiotherapy; *CT*, chemotherapy; *CRT*, chemoradiotherapy; *L-Asp*, L-asparaginase.

### 3.2 Risk construction and survival analysis

The cut-off values of inflammatory biomarkers are listed in Table S1. Higher PNI (P< 0.001), LMR (P= 0.002), ALB (P< 0.001), LYM (P= 0.003), and PLT (P= 0.027) were associated with better OS. Inflammatory biomarkers with P< 0.05 were included in the LASSO Cox regression analysis to construct the risk score. The LASSO Cox regression model (Eq. (2)) was used to calculate each patient’s risk score. According to the optimal cut-off value for the risk score (-1.3747), all patients were divided into high- and low-risk groups.

(2) 𝑅𝑖𝑠𝑘=-0.14413712∗𝑃𝑁𝐼-0.58395279∗𝐿𝑀𝑅-0.75817193∗𝑎𝑙𝑏𝑢𝑚𝑖𝑛-0.0.08354624∗𝑙𝑦𝑚𝑝ℎ𝑜𝑐𝑦𝑡𝑒-0.63571867∗𝑝𝑙𝑎𝑡𝑒𝑙𝑒𝑡

**Figure 1.**
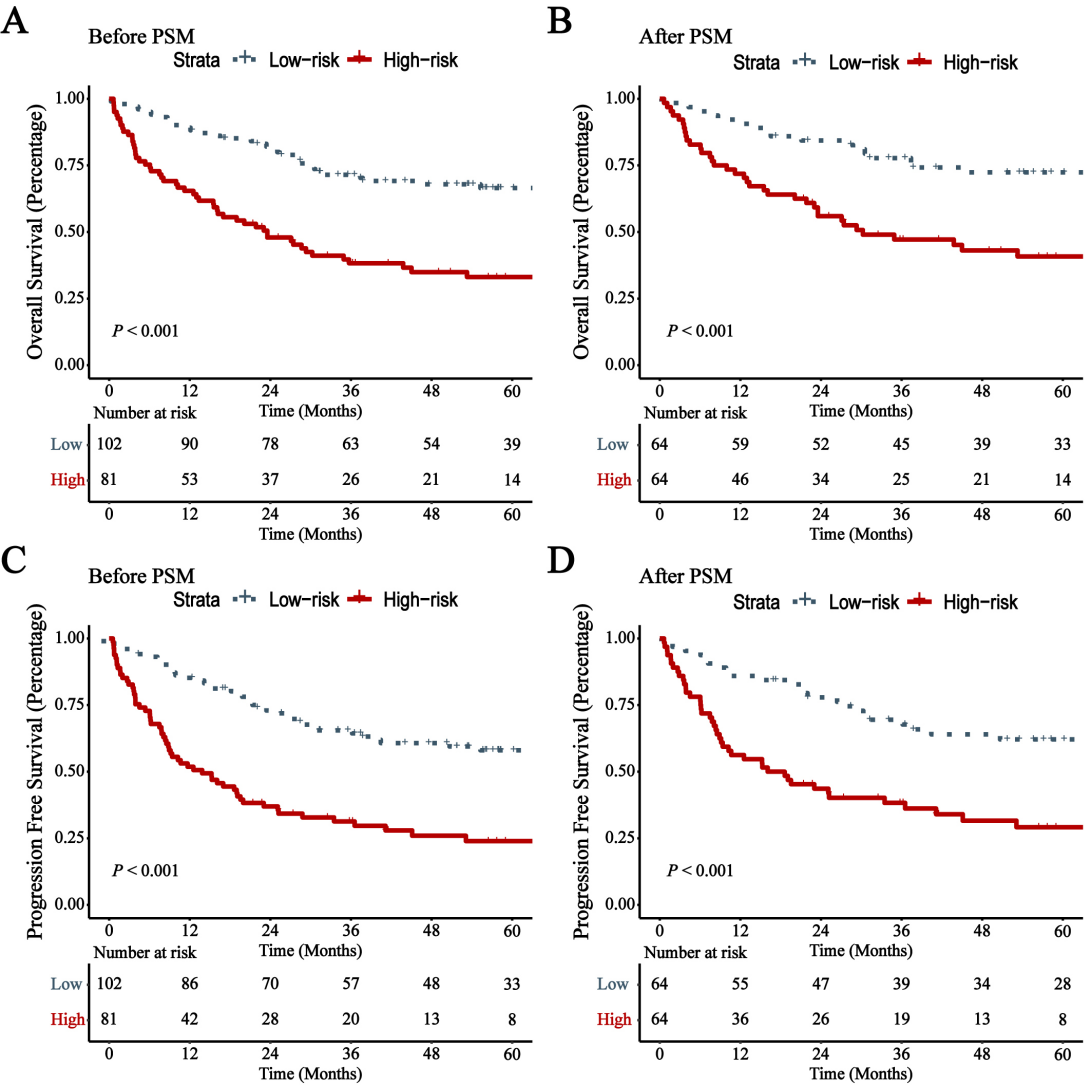
The survival curves of OS and PFS for risk groups before (A, C) and after PSM (B, D).

Before PSM, the 5-year PFS and OS rates in the high-risk group were 24.0% vs. 33.1%, respectively, whereas those in the low-risk group were 58.6% vs. 66.5%, respectively (Fig. 1). The baseline characteristics distribution between high risk and low risk has significant differences. To better control the bias caused by retrospective clinical data, the PSM method was applied to adjust the differences between variables so that all characteristics were well-balanced (Table 2). After PSM, the 5-year PFS and OS rates in the high-risk group were 29.2% vs. 40.8%, respectively, whereas those in the low-risk group were 62.1% vs. 72.4%, respectively (Fig. 1).

**Table 2:** Distribution of clinical characteristics of risk before and after PSM

Characteristics	Before PSM			After PSM		
	Low risk	High risk	P value	Low risk	High risk	P value
Age			0.439			0.214
⩽ 60	84 (82.35%)	63 (77.78%)		57 (89.06%)	52 (81.25%)	
> 60	18 (17.65%)	18 (22.22%)		7 (10.94%)	12 (18.75%)	
Gender			0.037			0.051
Male	86 (84.31%)	58 (71.6%)		55 (85.94%)	46 (71.88%)	
Female	16 (15.69%)	23 (28.4%)		9 (14.06%)	18 (28.12%)	
ECOG			< 0.001			0.626
0–1	93 (91.18%)	54 (66.67%)		55 (85.94%)	53 (82.81%)	
⩾ 2	9 (8.82%)	27 (33.33%)		9 (14.06%)	11 (17.19%)	
Ann arbor stage			0.023			0.536
I–II	85 (83.33%)	56 (69.14%)		50 (78.12%)	47 (73.44%)	
III–IV	17 (16.67%)	25 (30.86%)		14 (21.88%)	17 (26.56%)	
B symptoms			0.016			0.719
Yes	29 (28.43%)	37 (45.68%)		25 (39.06%)	27 (42.19%)	
No	73 (71.57%)	44 (54.32%)		39 (60.94%)	37 (57.81%)	
No. of extranodal sites			0.010			0.435
< 2	94 (92.16%)	64 (79.01%)		57 (89.06%)	54 (84.38%)	
⩾ 2	8 (7.84%)	17 (20.99%)		7 (10.94%)	10 (15.62%)	
LN			0.388			0.102
Yes	34 (33.33%)	32 (39.51%)		12 (18.75%)	20 (31.25%)	
No	68 (66.67%)	49 (60.49%)		52 (81.25%)	44 (68.75%)	
Primary site			0.653			1
UADT	95 (93.14%)	74 (91.36%)		59 (92.19%)	59 (92.19%)	
Non-UADT	7 (6.86%)	7 (8.64%)		5 (7.81%)	5 (7.81%)	
RT			0.002			1
Yes	78 (76.47%)	44 (54.32%)		41 (64.06%)	41 (64.06%)	
No	24 (23.53%)	37 (45.68%)		23 (35.94%)	23 (35.94%)	
L-Asp-based CT			0.706			0.215
No	57 (55.88%)	43 (53.09%)		26 (40.62%)	33 (51.56%)	
Yes	45 (44.12%)	38 (46.91%)		38 (59.38%)	31 (48.44%)	

*UADT*, upper aerodigestive tract NK/T-cell lymphoma; *LN*, Regional lymph node involvement; *RT*, radiotherapy; *L-Asp*, L-asparaginase; *CT*, chemotherapy.

Then, we used univariate and multivariate analyses to evaluate the prognostic value of the included factors. The results are shown in Table 2. Before PSM, multivariate analysis showed that Risk was significantly associated with OS (HR = 2.577, 95% CI = 1.614–4.114, P< 0.001) and PFS (HR = 2.679, 95% CI = 1.744–4.114, P< 0.001). After PSM adjustment, Risk was still an independent factor for OS (HR = 2.829, 95% CI = 1.601–5.001, P< 0.001) and PFS (HR = 2.877, 95% CI = 1.735–4.770, P< 0.001) (Table 3).

**Table 3:** Univariate and multivariate Cox analyses for OS before and after PSM

Characteristics	Overall survival							
	Before PSM				After PSM			
	Univariate analyses		Multivariate analyses		Univariate analyses		Multivariate analyses	
	HR (95% CI)	P value	HR (95% CI)	P value	HR (95% CI)	P value	HR (95% CI)	P value
Age	1.669 (1.027–2.712)	0.039	1.662 (1.019–2.711)	0.042	1.932 (1.017–3.672)	0.044	1.888 (0.985–3.618)	0.056
Gender	1.282 (0.784–2.098)	0.322			1.394 (0.760–2.559)	0.283		
ECOG	3.109 (1.971–4.905)	< 0.001	1.887 (1.129–3.154)	0.015	2.104 (1.125–3.936)	0.020	2.124 (1.127–4.004)	0.020
Ann arbor stage	1.505 (0.931–2.432)	0.095			1.417 (0.779–2.578)	0.254		
B symptoms	0.935 (0.600–1.458)	0.768			0.762 (0.440–1.320)	0.332		
No. of extranodal sites	1.644 (0.924–2.924)	0.091			1.182 (0.533–2.622)	0.680		
LN	0.628 (0.408–0.966)	0.034			0.565 (0.317–1.007)	0.053		
Primary site	1.027 (0.474–2.228)	0.945			1.254 (0.499–3.151)	0.630		
RT	0.483 (0.315–0.740)	0.001	0.578 (0.365–0.916)	0.020	0.553 (0.325–0.942)	0.029	0.528 (0.309–0.902)	0.019
L-Asp-based CT	0.557 (0.357–0.869)	0.010	0.501 (0.316–0.792)	0.003	0.635 (0.366–1.100)	0.105		
Risk	2.921 (1.886–4.524)	< 0.001	2.577 (1.614–4.114)	< 0.001	2.849 (1.616–5.022)	< 0.001	2.829 (1.601–5.001)	< 0.001
	**Progression free survival**							
Age	1.295 (0.814–2.061)	0.275			1.364 (0.731–2.545)	0.330		
Gender	1.336 (0.850–2.099)	0.209			1.388 (0.802–2.405)	0.242		
ECOG	2.662 (1.721–4.116)	< 0.001	1.532 (0.936–2.508)	0.090	1.824 (1.010–3.296)	0.046	1.605 (0.885–2.911)	0.120
Ann arbor stage	1.151 (0.723–1.834)	0.553			0.992 (0.558–1.761)	0.977		
B symptoms	0.953 (0.635–1.430)	0.817			0.767 (0.469–1.254)	0.291		
No. of extranodal sites	1.293 (0.734–2.277)	0.374			0.873 (0.399–1.911)	0.734		
LN	0.702 (0.471–1.047)	0.083			0.713 (0.415–1.224)	0.219		
Primary site	1.100 (0.555–2.180)	0.785			1.144 (0.494–2.647)	0.753		
RT	0.476 (0.322–0.704)	< 0.001	0.591 (0.386–0.903)	0.015	0.520 (0.323–0.836)	0.007	0.506 (0.313–0.816)	0.005
L-Asp-based CT	0.645 (0.433–0.960)	0.031	0.567 (0.377–0.853)	0.007	0.713 (0.439–1.157)	0.170		
Risk	2.911 (1.952–4.341)	< 0.001	2.679 (1.744–4.114)	< 0.001	2.837 (1.715–4.695)	< 0.001	2.877 (1.735–4.770)	< 0.001

*UADT*, upper aerodigestive tract NK/T-cell lymphoma; *LN*, Regional lymph node involvement; *RT*, radiotherapy; *L-Asp*, L-asparaginase; *CT*, chemotherapy.

**Figure 2.**
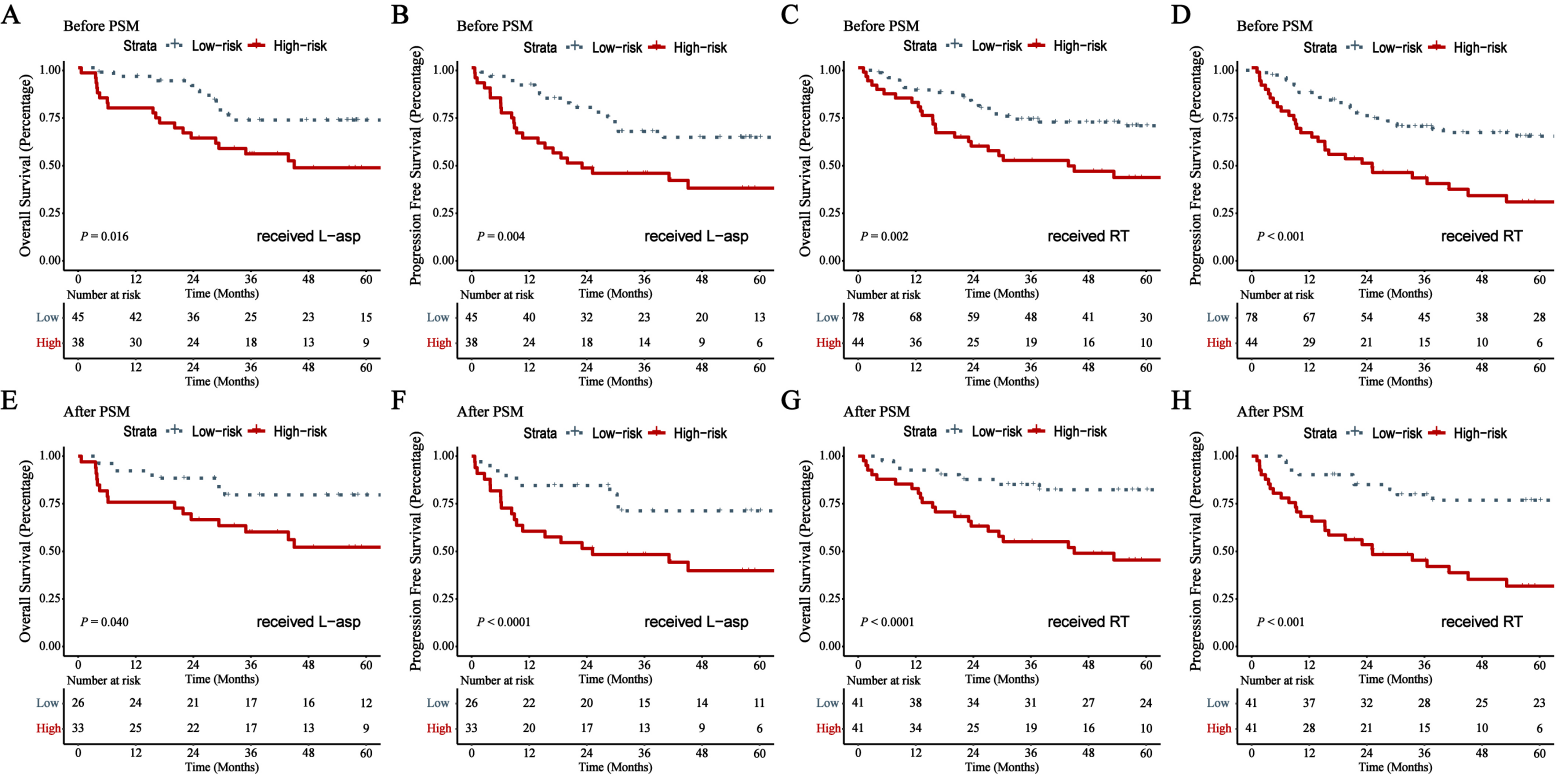
The survival curves of OS and PFS for high- and low-risk patients who received L-asp before (A, B) and after PSM (E, F); The survival curves of OS and PFS for high- and low-risk patients who received RT before (C, D) and after PSM (G, H).

**Figure 3.**
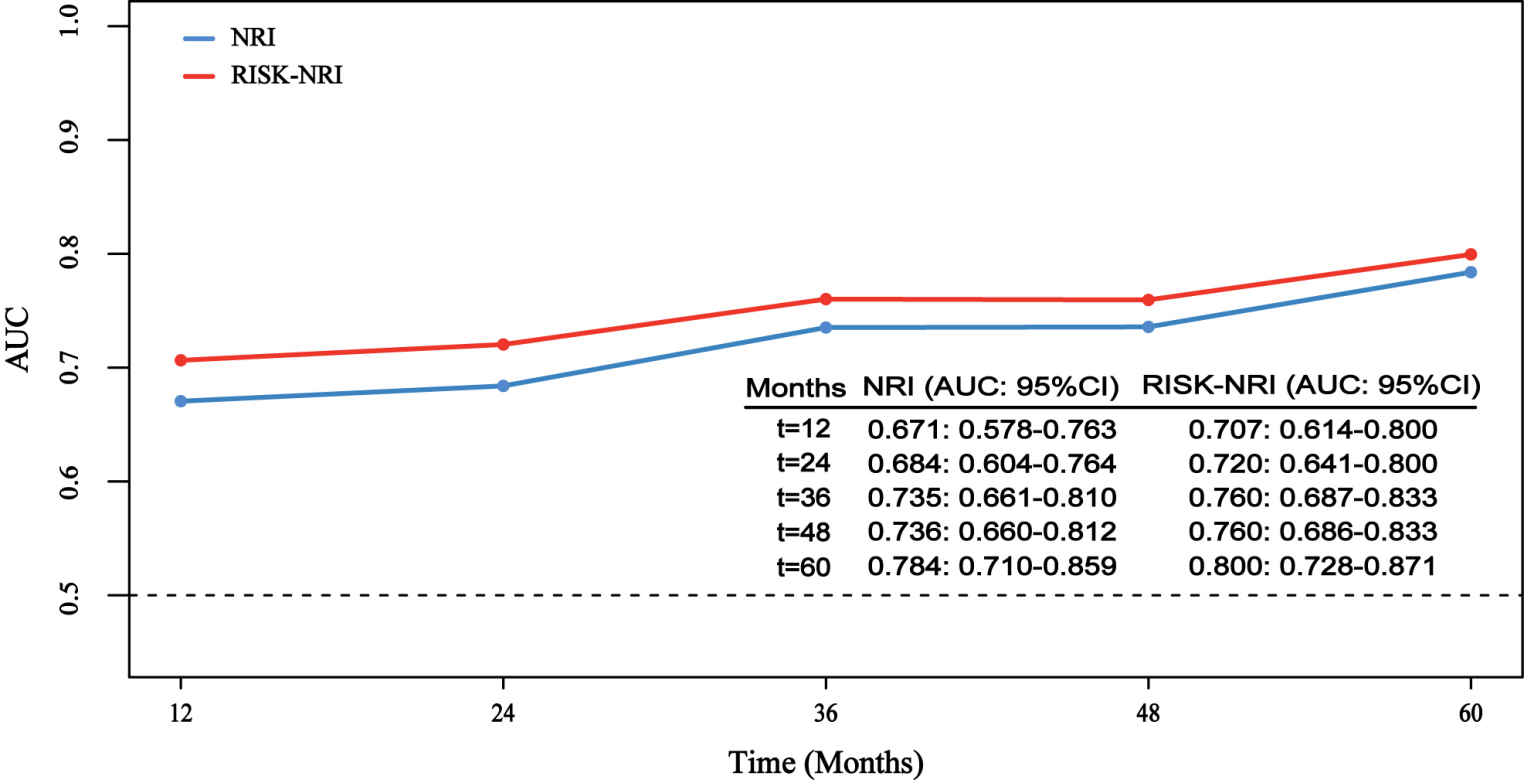
Time-dependent ROC curves of the NRI and RISK-NRI.

**Figure 4.**
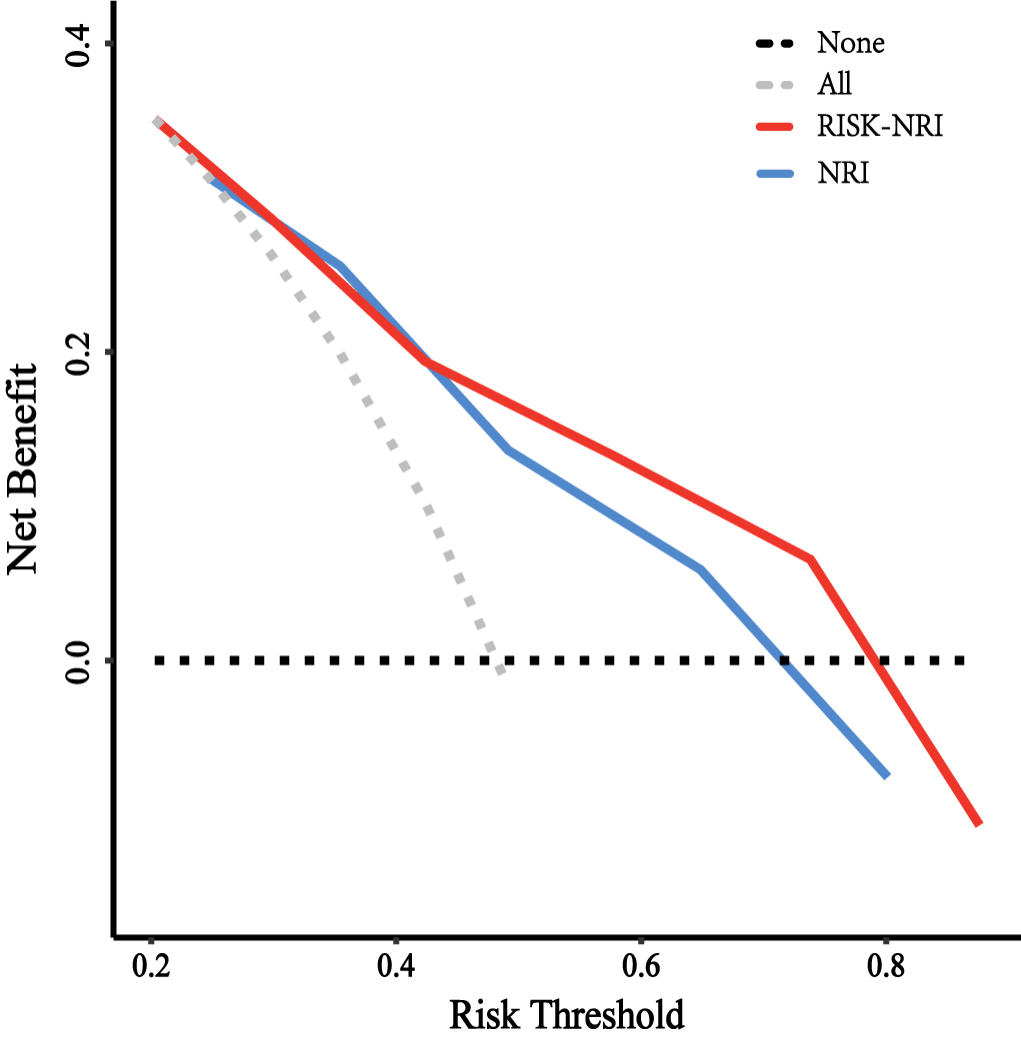
Decision curve analysis of the NRI and Risk-NRI.

### 3.3 Subgroup analysis

Before PSM, among 122 patients who received radiotherapy, the 5-year OS rates of the high- and low-risk groups were 42.4% and 69.6%, respectively (P= 0.002), and the 5-year PFS rates of the high- and low-risk groups were 29.6% and 65.0%, respectively (P< 0.001). Among the 83 patients who received L-ASP-based chemotherapy, the 5-year OS rates of the high- and low-risk groups were 47.5% and 72.5%, respectively (P= 0.016), and the 5-year PFS rates of the high- and low-risk groups were 36.8% and 63.5%, respectively (P= 0.004). After PSM adjustment, the 5-year OS rates of the high- and low-risk groups who received radiotherapy were 45.5% and 82.3%, respectively (P= 0.001), and the 5-year PFS rates of the high- and low-risk groups were 31.8% and 76.8%, respectively (P< 0.001). The 5-year OS rates of the high- and low-risk groups who received L-Asp-based chemotherapy were 52.1% and 79.6%, respectively (P= 0.016), and the 5-year PFS rates of the high- and low-risk groups were 39.8% and 71.3% (P= 0.010) (Fig. 2). Except for subgroups with a small number of patients, such as females, extranodal sites greater than 2, non-UADT, RT alone, and gemcitabine, other subgroup analyses showed OS and PFS differences between high-risk and low-risk groups (Fig. S1). 

### 3.4 Modified NRI by integrating risk

At 12, 24, 36, 48, and 60 months, the revised NRI predicted AUCs for OS of 0.707, 0.720, 0.760, 0.760, and 0.800, respectively, while the NRI predicted AUCs for OS of 0.671, 0.684, 0.735, 0.736, and 0.784, respectively (Fig. 3). The 5-fold cross-validation also showed that the AUC of the modified NRI in predicting the prognosis of ENKTCL at 0–60 months was higher than that of the NRI (Fig. S2). The decision curve showed that when predicting 5-year OS, the NRI had more clinical net benefit than “none” or “all” in the threshold probability from 0.246 to 0.801. In contrast, the modified NRI had a higher clinical net benefit in the threshold probability from 0.203 to 0.876 (Fig. 4). The IBSs in the NRI were 0.100, 0.139, 0.162, 0.173, and 0.179, respectively, and 0.096, 0.133, 0.154, 0.164, and 0.169 in the modified NRI, respectively (Fig. S3). The integrated discrimination improvement (IDI), continuous-net reclassification improvement (NRI), and median improvement in risk score were used to assess additional prognostic discrimination of the modified NRI (Table S2), and significant predictive performance improvements were observed compared with the NRI within 12, 24, 36, 48 and 60 months.

## 4. Discussion

ENKTCL is usually characterized by necrosis and inflammation. However, little is known about the effects of systemic inflammatory markers in ENKTCL. We used univariate Cox regression and LASSO Cox to integrate independent prognostic inflammatory markers proven by previous studies and constructed a comprehensive inflammatory index named Risk. We found that Risk was a prognostic factor for PFS and OS in ENKTCL patients. Moreover, the combination of Risk and NRI improves the performance of the NRI.

Systemic inflammation and malnutrition are important components of cancer [17, 18]. Malnutrition is responsible for nearly one-fifth of patients dying [19]. In addition to liver function, albumin is also one of the indicators of nutritional status and has been proven to be associated with the prognosis of various malignant tumours. Li et al. [20] found that albumin was independent of PFS (RR = 1.308, 95% CI: 1.037–1.649, P= 0.023) and OS (RR = 1.64, 95% CI: 1.284–2.094, P< 0.001). In this study, albumin was associated with OS and PFS, and patients with lower albumin had a poor prognosis. Lymphocytes can strengthen the immune surveillance ability and suppress the proliferation, invasion, and metastasis of tumour cells and have been used to predict the prognosis of patients with many types of tumours, such as oropharyngeal cancer, melanoma, and skin cancer [21, 22, 23, 24]. Serum monocyte levels can reflect the formation of tumour-associated macrophages (TAMs) in the tumour microenvironment [25]. TAMs can promote tumour initiation and growth by stimulating proliferation and angiogenesis, such as in primary hepatocellular cancer, gastric cancer, and neuroblastoma [26, 27, 28]. Lymphocytes and monocytes calculate the LMR ratio. As an inflammation- and immunity-related biomarker, it is an important prognostic marker for several tumours. Decreased LMR is considered to be closely related to Epstein-Barr virus DNA positivity, and the infection is closely related to the occurrence and development of ENKTCL [14, 29, 30]. Previous studies have found that decreased platelets are an adverse independent factor in diffuse large B-cell lymphoma, peripheral T-cell lymphoma, and ENKTCL [31, 32, 33, 34]. Our study also found that patients with low PLT levels had poor prognoses. However, how platelets affect the prognosis of ENKTCL patients is still unclear. Researchers have suggested that patients with low platelet levels in blood system diseases may have a higher tumour burden [34].

In our study, the Risk based on six inflammatory biomarkers effectively stratified OS and PFS in ENKTCL patients. High-risk patients benefit less from L-Asp-based chemotherapy and radiotherapy. Moreover, even if age, sex, extranodal site of involvement, ECOG score, Ann Arbor Staging System, B symptoms, regional lymph node involvement, primary site, and radiotherapy were adjusted by PSM analysis and multivariate analysis, the OS and PFS of patients with high risk were worse than those of patients with low risk. Furthermore, after combining Risk into the NRI, the modified NRI performance was improved, which can be demonstrated by AUC, IBS, and clinical net benefit. In the modified NRI performance at 12, 24, 36, 48, and 60 months, the AUC increased by 0.036, 0.036, 0.025, 0.024, and 0.016, respectively. IBS decreased by 0.004, 0.004, 0.008, 0.009, and 0.01, respectively. DCA shows that the modified NRI has favourable clinical application across a wider range of threshold probabilities. Adding Risk to the NRI also improved IDI, continuous NRI, and median improvement in risk score.

Although this study showed that integrating Risk could significantly improve the predictive performance of the NRI, there are still some limitations. First, this was a single-centre retrospective study with a small sample size and a lack of external validation. Second, because C-reactive protein was not counted, Glasgow’s prognostic score was not included in the analysis. Third, the optimal cut-off value of the inflammatory index was determined according to the maximally selected rank statistics method, which may be inconsistent between different centres. In conclusion, further multicentre large-scale studies are needed to confirm the conclusions of this study.

## 5. Conclusions

Risk is routinely available and inexpensive and can predict the prognosis of ENKTCL. Combining Risk and NRI can effectively improve the prediction performance. 

## Author contributions

Conception: Cao J.

Interpretation or analysis of data: Hou Q, Li H, Liang Y, Yao N, Cao X, Sun B, Feng P, Zhang W, Liu J.

Preparation of the manuscript: Hou Q, Li H, Yao N, Cao X, Liu J, Sun B, Feng P, Zhang W.

Revision for important intellectual content: Liu J, Cao J.

Supervision: Cao J.

## Funding

This research was funded by the Applied Basic Research Projects of Shanxi Province [No. 20210302124598], Wu Jieping Medical Foundation No. 320.6750.2022-1-53, Lianyungang Yixing Medical Health Foundation, the Research Project Supported by Shanxi Scholarship Council of China No. [2022]210, and the Four “Batches” Innovation Project of Invigorating Medical through Science and Technology of Shanxi Province No. 2022XM32.

## Institutional review board statement

This study was approved by the Ethics Committee of Shanxi Province Cancer Hospital, Shanxi Hospital Affiliated to Cancer Hospital, Chinese Academy of Medical Sciences, Cancer Hospital Affiliated to Shanxi Medical University, and the review committee exempted informed consent requirements on 3 September 2019. (Approval code 2019091).

## Informed consent statement

Written informed consent was waived due to the retrospective study design.

## Data availability statement

The datasets generated and analysed during this study are not publicly available due to ethical conditions but are available from the corresponding author on reasonable request. 

## Supplementary data

The supplementary files are available to download from http://dx.doi.org/10.3233/CBM-230067.

## Supplementary Material

Supplementary tables 1-2
