# Supplementary material for: Impact of the peripheral blood inflammatory indices and modified nomogram-revised risk index on survival of Extranodal Nasal-Type Natural Killer/T-Cell lymphoma
Source: Cancer Biomark. 2024 Jan 5;39(1):27–36. doi: 10.3233/CBM-230067 (PMC10977361; doi:10.3233/CBM-230067)
Supplement: Supplementary tables 1-2 [file cbm-39-cbm230067-s001.docx]

**Table S1.** Univariate Cox analysis for OS of inflammation-related factors in ENKTCL.

|  | **Cut point** | **Statistic** | **HR (95%CI)** | ***P* value** |
| --- | --- | --- | --- | --- |
| Albumin | 37.3 | 4.097246 | 0.392(0.254-0.605) | <0.001 |
| Monocyte | 0.64 | 1.944092 | 1.613(0.966-2.692) | 0.068 |
| Lymphocyte | 1.41 | 3.340931 | 0.531(0.347-0.812) | 0.003 |
| Platelet | 144 | 2.172362 | 0.531(0.303-0.929) | 0.027 |
| NLR | 1.065693 | 1.299225 | 0.734(0.414-1.301 | 0.290 |
| dNLR | 2.545455 | 1.309414 | 1.368(0.794-2.356) | 0.259 |
| PLR | 156.3758 | 1.884168 | 1.448(0.947-2.214) | 0.087 |
| LMR | 3.909091 | 3.479470 | 0.477(0.302-0.754) | 0.002 |
| PNI | 48.8 | 3.903493 | 0.447(0.289-0.693) | <0.001 |

**Table S2.** The IDI, Continuous-NRI, and Median improvement were used to assess reclassification performance and improvement in discrimination of RISK-NRI.

|  | **IDI (95%CI)** | | **Continuous-NRI (95%CI)** | | **Median improvement in risk score (95%CI)** | |
| --- | --- | --- | --- | --- | --- | --- |
|  | **Point** | ***P* value** | **Point** | ***P* value** | **Point** | **P value** |
| 1-year | 0.034(0.007-0.064) | 0.004 | 0.347(0.178-0.487) | <0.001 | 0.061(0.033-0.104) | <0.001 |
| 2-year | 0.048(0.016-0.078) | <0.001 | 0.366(0.218-0.498) | <0.001 | 0.093(0.047-0.141) | <0.001 |
| 3-year | 0.049(0.011-0.080) | 0.012 | 0.341(0.188-0.471) | <0.001 | 0.117(0.038-0.151) | <0.001 |
| 4-year | 0.048(0.010-0.081) | 0.016 | 0.337(0.184-0.476) | 0.004 | 0.119(0.037-0.151) | <0.001 |
| 5-year | 0.049(0.007-0.085) | 0.032 | 0.348(0.176-0.511) | 0.004 | 0.119(0.020-0.151) | 0.004 |
